# Zinc oxide nanoparticles mitigate insulin resistance in a D-galactose-induced C57BL/6 mouse model

**DOI:** 10.3389/fendo.2026.1819985

**Published:** 2026-05-12

**Authors:** Kanagavalli Ramasubbu, Devi Rajeswari V.

**Affiliations:** Department of Biomedical Sciences, School of Biosciences and Technology, Vellore Institute of Technology, Vellore, Tamil Nadu, India

**Keywords:** amyloid-β deposition, insulin resistance, insulin signalling, neurodegeneration, ZnO nanoparticles

## Abstract

D-galactose promotes insulin resistance (IR) and neurodegeneration through hyperglycemia, advanced glycation end products (AGEs), oxidative stress, amyloid-β accumulation, and disruption of the insulin signalling pathway, leading to neuroinflammation and neuroapoptosis. The purpose of this study was to investigate the insulin signalling pathway and efficacy of ZnO nanoparticles (ZnO NPs) in alleviating brain IR and neurodegeneration via regulating IRS/PI3K/AGER/APP signalling. In this study, bioinformatic analysis of hub genes and PIP network revealed the interconnection between PI3K/Akt signalling pathway and neuroinflammation pathway leading to neurodegeneration. Further, *in vivo* analysis on 4-month-old C57BL/6 mice was randomised into control (NC), 50 mg/kg of D-galactose (DC), D-galactose + 450 μg/Kg of ZnO NPs (Z1), D-galactose + 650 μg/Kg of ZnO NPs (Z2), and D-galactose + 20 mg/kg of metformin (MF). Blood glucose, Amadori products, conjugated dienes, amyloid-β deposition, and gene and protein expression of the IRS/PI3K/AGER/APP signalling pathway were analysed. Our findings illustrated that ZnO NPs (Z1) alleviated hyperglycemia, cleared amyloid-β accumulation, maintained neural integrity, lessened fat deposition in the liver, and altered the expression of *irs2*, *pi3k*, *ager*, and *app* genes. Thus, ZnO NPs offer a therapeutic strategy for managing IR and neurodegeneration.

## Graphical Abstract

**Figure:**
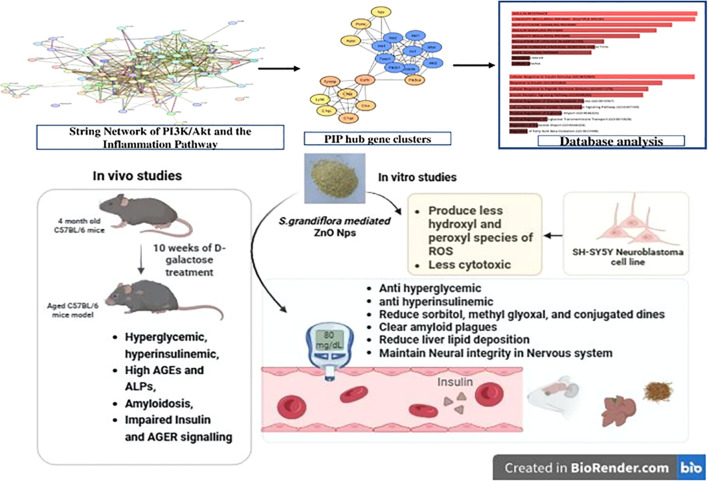
The abstract should ideally be structured according to the IMRaD format (Introduction, Methods, Results and Discussion). Provide a structured abstract if possible. If your article has been copyedited by us, please provide the updated abstract based on this version.

## Highlights

- IR and ageing are major factors for the progression of neurodegeneration.
- Zn is crucial in treating numerous diseases, including IR and neurodegeneration.
- PI3K/Akt/AGER signalling pathway can promote amyloid deposition in IR and lead to neurodegeneration
- ZnO NPs alleviate hyperglycemia, hyperinsulinemia, amyloid plaque accumulation, deposition of ALPs, and amadori products.
- Further, maintained the *PI3K* signalling, *rage*, and *app* expressions.

## 1 Introduction

Metabolic syndrome, like insulin resistance (IR), hyperglycaemia, diabetes, dyslipidaemia, neurodegenerative diseases, non-alcoholic fatty liver (NAFLD), obesity, hypertension, and cardiovascular diseases, causes a significant public health challenge (1). Metabolic imbalance disrupts mitochondrial function, antioxidant defence mechanism, immunity, glucose uptake, protein synthesis, and protein misfolding. In the brain, it causes neuroinflammation, cognitive dysfunction, and motor impairment (2–4). IR contributes to the synthesis of Advanced glycation end products (AGEs) and Advanced lipid peroxidation products (ALPs), which influence cellular and metabolic functions, Lewy body accumulation, oxidative stress, neuroinflammation, and neural signalling, leading to progressive neurodegeneration (5). As seen in diseases like Alzheimer’s disease (AD), Parkinsonvalign="top"’s disease (PD), dementia, Huntington’s disease (HD), and Amyotrophic lateral sclerosis (ALS). Ageing, nutritional deficiencies (Zn, Fe, Ca, Mg, Mn, and vitamins), heredity, and environmental factors may contribute to neurodegeneration through chronic inflammation, Blood-Brain Barrier (BBB) disruption, mitochondrial dysfunction, and protein accumulation (amyloid-β, tau, α-synuclein, and TDP-43) (6, 7).

Zn, Fe, Mg, Mn, Se, and Cu are crucial for insulin sensitivity by regulating insulin receptor function, oxidative damage, and inflammation (8, 9). Zn deficiency affects the synthesis, secretion, storage, and structural integrity of insulin, subsequently worsening IR. Zn supplementation maintains insulin secretion, synaptic function, neural plasticity, cell proliferation, and metabolic homeostasis through the *PI3K*/Akt signalling pathway (1, 10, 11). However, excess Zn leads to pancreatitis, IR, mitochondrial dysfunction, amyloid-β aggregation, oxidative stress, and disruption of Ca^2+^ homeostasis in neurons, resulting in neuronal apoptosis (12–14).

Disruption of the Insulin and RAGE signalling pathways, particularly insulin-mediated PI3K/Akt, Ras/MAPK signalling, AGE-RAGE, ALP-RAGE, ALP-LOX 1, and ALP-neutrophil PAF receptor interactions, triggers proinflammatory cytokines (IL-6 and TNF-α), ROS production, endothelial dysfunction, protein accumulation (amyloid-β and synuclein), and autophosphorylation (15, 16). Like Zn, ZnO NPs, due to their stability, a high surface area, targeted delivery, controlled release, support the synthesis of neurotransmitters (glutamate, dopamine, acetylcholine, and GABA), enhance expression of neurotrophic factors (BDNF), promote the activation of antioxidant enzymes (SOD), and protect pancreatic functions (17). The green synthesis of NPs is a sustainable, environmentally friendly, biocompatible, minimally cytotoxic, cost-effective, scalable, and reproducible process (18). Hence, ZnO NPs were fabricated using *Sesbania grandiflora*, a widely used edible plant in Indian traditional medicine. It exhibits antioxidant, antimicrobial, wound-healing, antidiabetic, neuroprotective, and anticancer effects. In our previous study, anti-oxidant, antidiabetic, anti-Amadori, and cytotoxic activities of *S. grandiflora-*mediated ZnO NPs were analysed (19). The current study aims to investigate the role of the insulin signalling pathway in inflammation and neurodegeneration and the therapeutic effects of *S. grandiflora*-mediated ZnO NPs in D-galactose-treated C57BL/6 mouse model. Mainly, focusing on hyperglycaemia, neurodegeneration, and insulin signalling regulation.

## 2 Materials and methods

### 2.1 Transcriptomic data collection and analysis strategies

Microarray datasets GSE152539 (12 samples, diet induced diabetic AD model), GSE132940 (192 Samples, metabolic hormone signalling in brain), GSE280980 (10 Samples, brain inflammation) GSE156762 (9 Samples, mTOR signalling in ageing brain), GSE179711 (8 Samples, Diet induced diabetes and brain), GSE106903 (43 samples, diet induced insulin signalling), and GSE237891 (13 samples, neuroinflammation induced neurodegeneration) were retrieved from GEO database (https://www.ncbi.nlm.nih.gov/geo/). Visual and interactive Differential expression GEO2 (DEG) analysis was performed using the web *app*lication Phantasus (https://artyomovlab.wustl.edu/phantasus/) with statistical significance (p≤ 0.05 and a LogF ≥ 1). Further analysis was performed in Enrichr (https://maayanlab.cloud/Enrichr/) to assess the GO ontology, KEGG, and Protein-Protein Interaction (PIP). Visualisation was done in String network (https://string-db.org/ and the Cytoscape plugin. Unm*app*ed genes in the datasets were identified and converted with the Gene Id conversion tool David (https://davidbioinformatics.nih.gov/) (20, 21).

### 2.2 Synthesis and characterisation of ZnO NPs

The synthesis and characterisation of ZnO NPs were mentioned in our previous publication (19). Here, further analysis of ZnO NPs using Atomic force microscopy (AFM), Zeta potential, and Dynamic light scattering (DLS) was included. For Zeta potential and DLS, ZnO NPs were sonicated with PBS (with viscosity 0.893 mPa*s, conductivity 0.125 mS/cm for 5 mins and measured at a temperature of 25.1 °C, conductivity 0.125 mS/cm, and electron Voltage 3.4 V.

### 2.3 *In vitro* analysis of ZnO NPs

SH-SY5Y human neuroblastoma cell lines were procured from ATCC (ATTC CRL-2266), cultured in DMEM-F12 hymidine with 10% inactivated Fetal Bovine Serum (FBS), penicillin (100 IU/mL), and streptomycin (100µg/mL) in a humidified atmosphere of 5% CO2 at 37 °C. ZnO NPs (320 µg/ml, 160 µg/ml, 80 µg/ml, 40 µg/ml, 20 µg/ml, 10 µg/ml, and 5 µg/ml) were prepared in DMEM media by sonication. Doxorubicin (25 mM) was used as a standard drug.

### 2.4 MTT analysis

100 µL of ZnO NPs and Doxorubicin at various concentrations were added to a 96-well plate, with SH-SY5Y cells for 24 hrs of incubation. After incubation, add 100 µL of MTT reagent, incubate for 4 hrs at 37 °C in 5% CO^2^. The supernatant was removed, and 100 µL of DMSO was added (22, 23). The absorbance was measured using a microplate reader at 590nm in a multimode plate reader, and the percentage growth inhibition was calculated with

% of inhibition = (OD of control-OD of sample)**/**(OD of control) * 100

### 2.5 ROS generation analysis with DCFHCA method

Assay performed with DCFDA**/**H2DCFDA - Cellular ROS Assay Kit following the given protocol. In a 96-well plate, 100 µl of the SH-SY5Y cells was incubated for 24 hrs. Then, 100µl of ZnO NPs (40 and 80 μg/ml) and doxorubicin (25 μM) were added, and the plates were incubated at 37 °C/5% CO^2^ atmosphere for 24 hrs. After washing the cells with 1X PBS, 100µl of 25µM DCFDA solution was added and incubated for 30 mins. The fluorescence was measured using a microplate reader at an excitation and emission maxima of 485/535 nm.

% of ROS generation = (OD of sample/control X 100)-100

### 2.6 Apoptotic assay with FITC, annexin V, and PI staining with flowcytometry

SH-5YSH cells (1 × 10^6^) were treated with ZnO NPs (40 and 80 μg/ml) and doxorubicin (25 μM) and incubated for 24 hrs. The cells were detached using a 0.05% trypsin-EDTA solution, transferred to RIA tubes, and centrifuged at 4000 rpm for 5 mins at 4 °C. The collected pellets were washed twice and resuspended in 2 ml of cold 1X PBS. Then, 500 μl of cell suspension was stained with 1 µl of Propidium Iodide (PI) and 5 μl of Annexin V and incubated in the dark for 15 mins at room temperature. After incubation, the cells were immediately analysed with a flow cytometer using FL1 and FL2 filters and CellQuest software (24, 25).

### 2.7 Randomisation and D-galactose ingestion in C57BL/6 mice

30 C57BL/6 mice were *app*roved for the study by the Institutional Animal Ethical Committee (IAEC), Vellore Institute of Technology- Vellore, Tamil Nadu, India (Ethical number – VIT/IAEC/21/Sep22/11). The mice were randomised into 5 groups: control (NC), disease control (DC), 450 μg/Kg ZnO NPs (Z1), 650 μg/Kg ZnO NPs (Z2), and 20 mg/Kg metformin (MF). Except for NC, all other groups were treated with D-galactose (50 mg/Kg) for 10 weeks and a normal chow diet throughout the study. The body weight of each group was measured from the 1st to the 4th month, and after the treatment. Mice were anaesthetised with 3-4% isoflurane for induction, maintained at 1-1.5% isoflurane in oxygen (0.5-1L/min) during the experimental procedure.

Fasting blood glucose (FBG) and oral glucose tolerance test (OGTT) were performed using a glucometer (One Touch Verio version) after mice had fasted for 6 hrs. For OGTT, followed by 6 hrs fasting, mice were fed with 0.5 ml of glucose (6 g/10 ml). Then the blood glucose was measured at 30 mins intervals, and the values were plotted in mg/dl.

### 2.8 Inductive coupled plasma mass spectroscopy analysis

From each group, 100 mg of brain, liver, and pancreas were collected, thermally digested, homogenised, treated with a few drops of nitric acid, and made up to 5 ml with ultrapure water. For the blood sample analysis, 0.1 ml of whole blood from all groups was diluted with 4 ml of ultra-pure water and 25 μl of nitric acid, then made up to 5 ml. These prepared tissues and blood are used for Inductive Coupled Plasma Mass Spectroscopy (ICP-MS) analysis, and the final concentration is calculated (26).

Final concentration (µg/g) = Unfactored concentration (µg/L)× Final Volume (L)/Sample weight (g)

### 2.9 Methylglyoxal analysis

The presence of methylglyoxal was measured through the addition method. Whole blood (200 μl) was mixed with an equal volume of acetonitrile and methylglyoxal (100 mmol/ml). Then, methylglyoxal was measured using a UV spectrophotometer at wavelengths ranging from 200 to 500 nm (27).

### 2.10 Sorbitol analysis

The RBC was homogenised with 0.85% sodium hydroxide for 5 mins. Then, 10% trichloroacetic acid (800 μl) was added, and the mixture was centrifuged at 6000 rpm for 15 mins. With the collected precipitate, an equal volume of 250 mmol/l sodium carbonate was added. 70 μl of this mixture, 20 μl of potassium dihydro phosphate (1 mol/l), 3 μl of benzoyl chloride, and 15 μl of sodium hydroxide (8 mol/l) were added and mixed at a vibrator mixture (2000 rpm). Then, 75 μl of ethyl acetate was added and vortexed, and the ethyl acetate phase was collected and dried in a vacuum desiccator till it reached 25 μl, acetonitrile and water mixture (80:20) was added (28). This mixture was analysed with a UV spectrophotometer (Jasco V 750) (200-400nm). Here, Milli-Q water was used as a blank. Sorbitol, glucose, and galactose were used as standards.

### 2.11 Estimation of conjugated dienes

Brain and liver tissues (100 mg) were homogenised, extracted with chloroform: methanol (2:1), and centrifuged at 2000 rpm for 10 mins. The chloroform phase was collected and dried at 45 °C. The dried residue was reconstituted in n-hexane and measured at 260 nm in UV-visible spectroscopy. N-hexane was used as a blank (29).

### 2.12 Insulin analysis with HPLC

PBS (pH 7.4) diluted plasma (0.2 ml) was vortexed for 1 min with 1 ml of dichloromethane and centrifuged at 2000 rpm/3 min. Then, the organic phase was transferred to fresh tubes and vortexed with 0.05M of hydrochloric acid. The supernatant was evaporated at room temperature. Then, residues were dissolved in PBS (pH 7.4) and analysed using HPLC at 280 nm and a UV spectrophotometer (Jasco V 750) between 200–400 nm (30, 31). Insulin was used as a standard phosphate buffer as a blank.

### 2.13 Histopathological analysis

#### 2.13.1 Hematoxylin & eosin staining

Liver tissues of NC, DC, Z1, Z2, and MF were deparaffinised with xylene and rehydrated with a gradient of ethanol 100%, 90%, and 80%. Then, the tissue slice was fixed on a slide and stained with Hematoxylin for 30 seconds, washed with PBS (pH 7), and counterstained with eosin. The slides were dried, covered with coverslips, and observed under a light microscope (32).

#### 2.13.2 Congo red staining

Specimens (NC, DC, Z1, Z2, and MF) were sliced, deparaffinised with xylene and rehydrated with an alcohol gradient of 100%, 90%, and 70%. Then it was stained with Congo red (prepared in 80% ethanol) and sodium chloride. The slides were dried, covered with coverslips, and observed under a light microscope. Total area of amyloid beta expression was analysed with ImageJ (33).

#### 2.13.3 Nissl staining

The brain and spinal cord tissues (5mm) were washed with xylene and ethanol gradients of 100%, 95%, and 80% for 30 seconds each, then stained with cresyl violet solution for 2 mins. Then the slides were covered with a coverslip, and the neurons were counted using ImageJ (34).

#### 2.13.4 Immunohistochemistry

Expression of *PI3K* and *AGER* in NC, DC, Z1, Z2, and MF was analysed. Deparaffinised fixed tissue slices were incubated in trypsin-PBS solution for 10 min at 37 °C. 100 μl of the diluted primary antibodies *AGER* Rabbit mAb (A23422) (1:5000 dilution) and *PI3K* Rabbit mAb (A4992) (1:60 dilution) was added to the *app*ropriate slides and incubated at 37 °C/60 mins. Followed by, the slides were washed with PBS. Conjugated secondary antibody Goat Anti-Rabbit IgG (H+L), which were diluted in peroxidase (1:5000), were added to the slides and incubated at 37 °C/30 mins, followed by washing with PBS for 5 mins and observed under the microscope.

### 2.14 Gene expression analysis

RNA was isolated from brain tissues using the Trizol method, and mRNA was quantified using a Nanodrop. From the RNA, cDNA was synthesised using a Bio-Rad iScript cDNA synthesis kit as per the instructions. Primers were diluted 1:1000 with Tris-EDTA buffer. The gene sequences are given in **Table 1**. The obtained cDNA was analysed with agarose gel electrophoresis.

**Table 1:** DNA primer sequence.

S.No	Gene	Sequence
1	*irs 2*	Fr CCAGTAAACGGAGGTGGCTACA Rv CATAGACAGCTTGGAGCCACA
	*pi3k*	Fr GAAGCACCTGAATAGGCAAGTCG Rv GAGCATCCATGAAATCTGGTCGC
3	*ager*	Fr GCCACTGGAATTGTCGATGAGG Rv GCTGTGAGTTCAGAGGCAGGAT
4	*app*	Fr TCCGTGTGATCTACGAGCGCAT Rv GCCAAGACATCGTCGGAGTAGT
5	*gadph*	Fr GTCTCCTCTGACTTCAACAGCG Rv ACCACCCTGTTGCTGTAGCCAA

### 2.15 Statistical analysis

G power 3.1.9.7 was used for analysing sample size to ensure the robustness and reproducibility of a predicted large effect size (Cohen’s d = 0.5), α level 0.05, and a desired power of 0.80. The minimum total sample size required to find a significant difference between the groups was determined as n=26. To ensure the statistical validity of parametric testing, the total n =30 animals were utilised (n=6 per group), which exceeds the actual power of 0.80.

All data analysis was performed in Python (3.13.64-bit version) using pandas, SciPy, Statsmodels, seaborn libraries, and Matplotlib. One-way analysis of variance (ANOVA) and two-way ANOVA followed by *post hoc* (TUKEY test) were *app*lied to determine the significant difference between the groups. Results are presented as mean ± standard error, and a p-value <0.05 was considered statistically significant. Additional figures were generated and processed using ImageJ 1.54p, Biorender (https://www.biorender.com/), and Origin Pro 2023. IC_50_ calculations were performed using GraphPad Prism (Version 8).

## 3 Results and discussion

### 3.1 Enrichment analysis and PIP hub gene analysis

**Figure 1:**
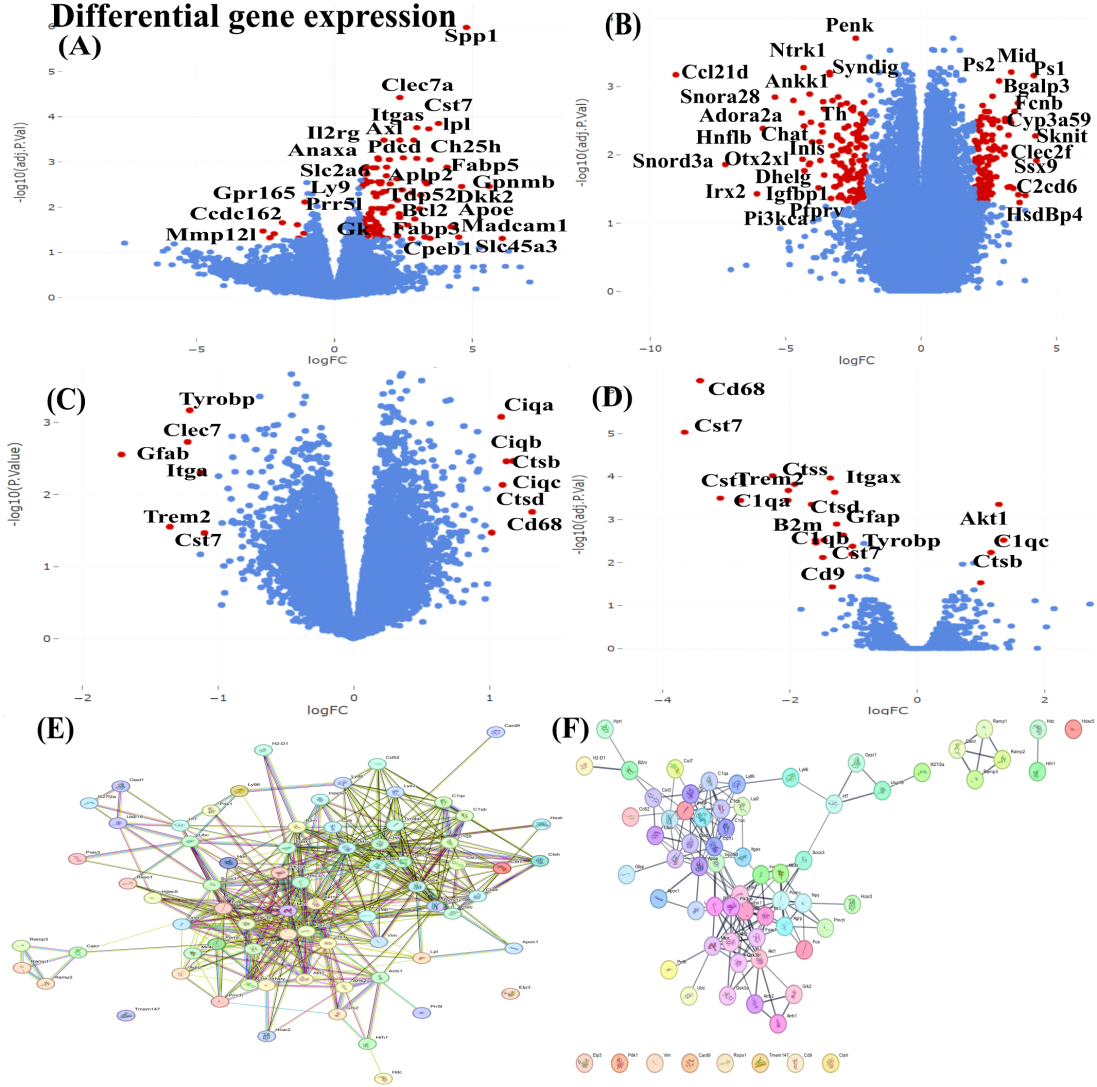
Volcano plot and string network m*app*ing of identified genes. **(A–D)** Shows an interactive scatter plot displaying Log2 fold changes and statistical significance calculated by differential gene expression analysis. Each point represents a gene; red- significant, and blue- non-significant. **(E)** String network of selected genes through Enrichment analysis **(F)** 72 identified clustered genes.

Datasets GSE152539, GSE132940, GSE280980, GSE156762, GSE179711, GSE106903, and GSE237891 were used for DEG analysis using limma (**Figures 1A–D**). The DEGs were defined by logFC ≥ 1 and p < 0.05, yielding 967 genes, including 172 overl*app*ing genes (**Figure 1E**). Overlapping genes were selected irrespective of the direction of regulation based on a recurrence ≥ 3 across more than three datasets. Of these, 72 were filtered using a STRING database (confidence score ≥ 0.7) based on database evidence, expression, and co-expression data (**Figure 1F**).

**Figure 2:**
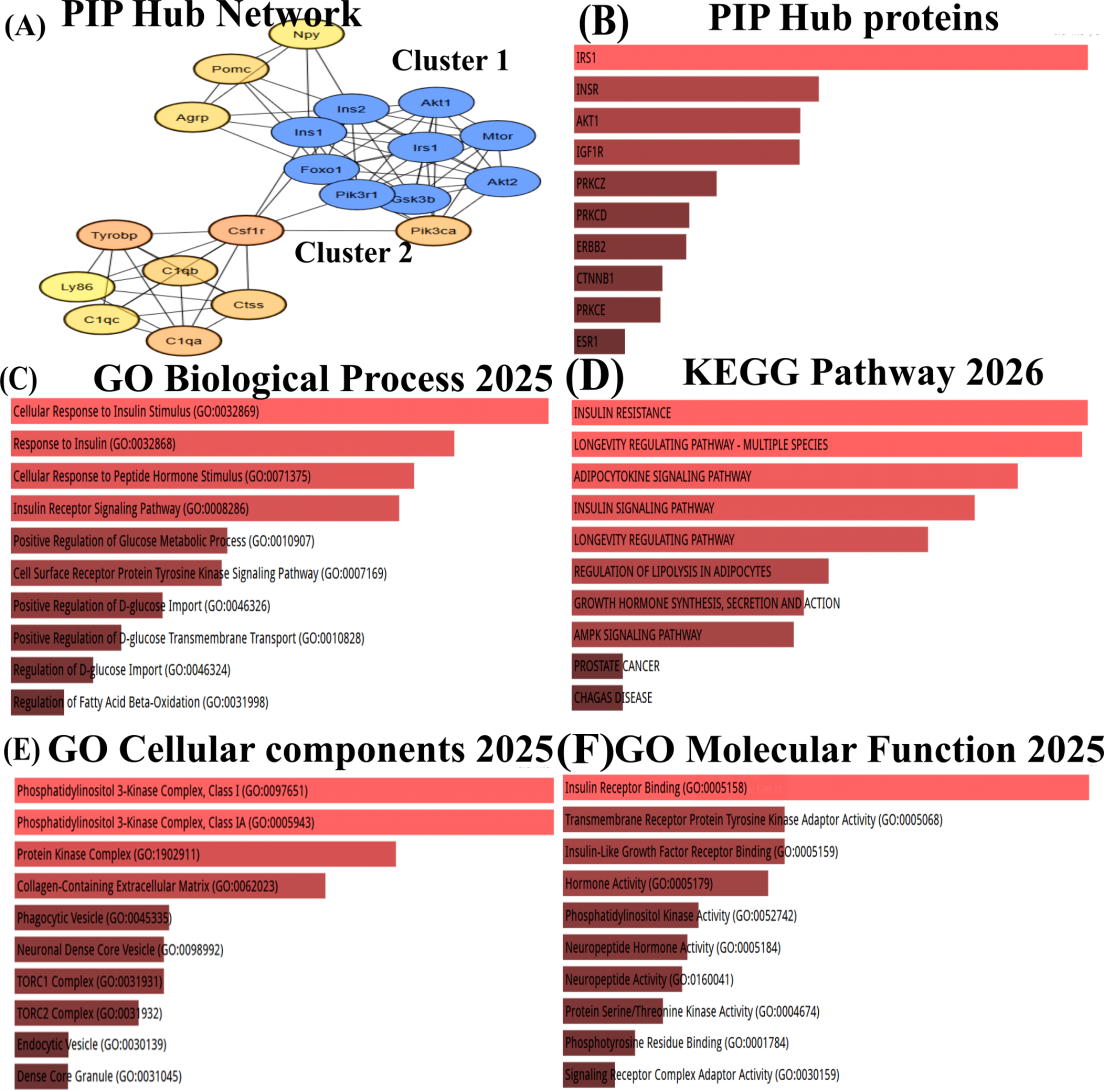
**(A)** Protein-protein interaction network clusters **(B)** Enrichment identified PIP hub proteins **(C)** Gene ontology -biological process of cluster genes **(D)** KEGG pathways mediated by cluster genes **(E)** Gene ontology - cellular components of clusters **(F)** Gene ontology- molecular functions of cluster genes.

**Figure 3:**
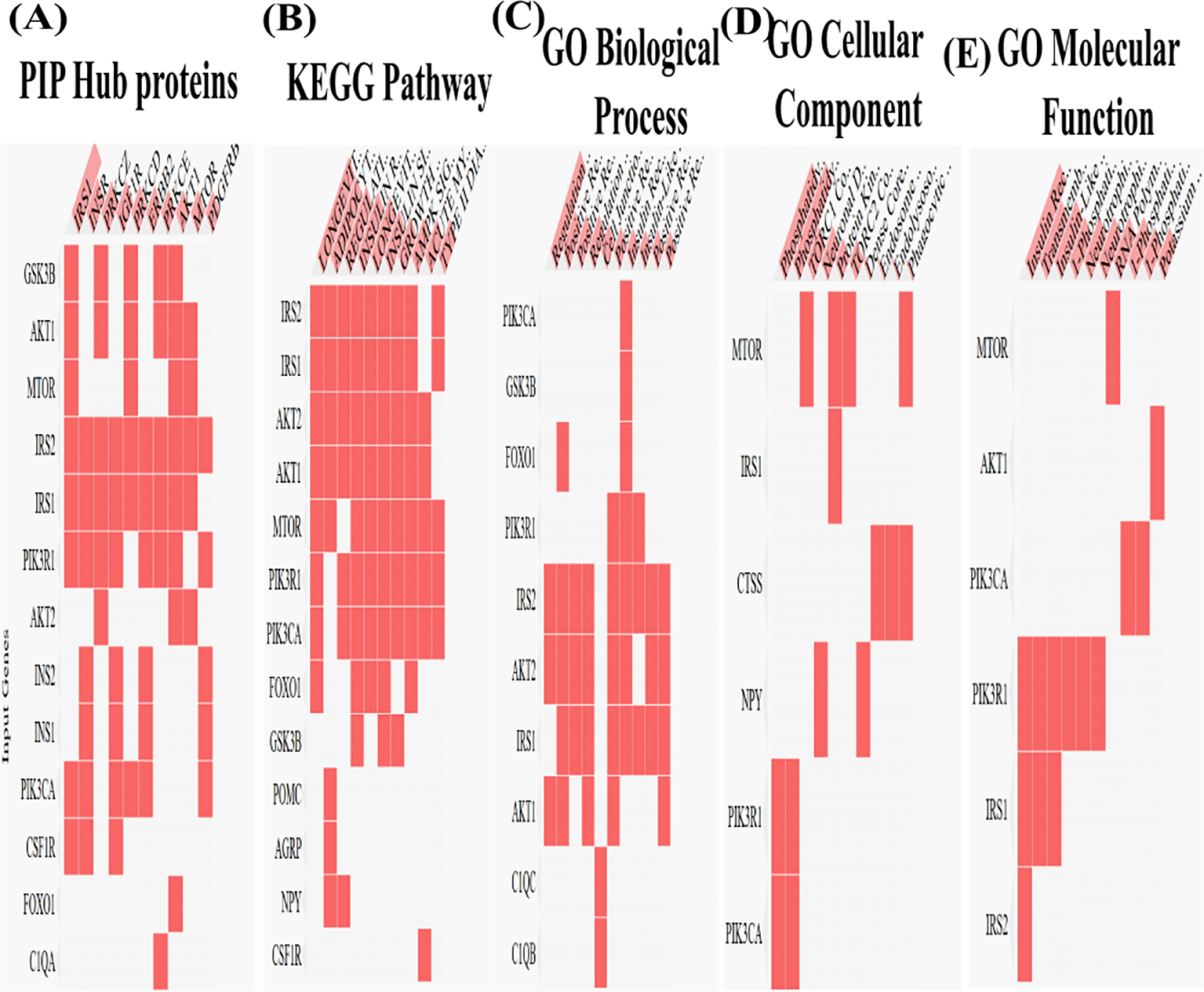
Gene interaction heat map of cluster genes **(A)** Protein-protein interaction, **(B)** KEGG network, **(C)** Gene Ontology -Biological process of cluster genes, **(D)** Gene Ontology - Cellular components of clusters, **(E)** Gene Ontology- Molecular functions of cluster genes.

The STRING network was then exported to Cytoscape, where the top 20 were identified as hub genes through CytoHubba, and gene clusters (cluster 1 and cluster 2) were determined with the scores of 8.5 and 6.6 each respectively (**Figure 2A**) (**Table 2**), revealing interconnected functional nodules between the *PI3K*/Akt signalling pathway, microglial activation, and the inflammation pathways in the diabetic brain (**Figures 2B–F**)(**Supplementary Tables 1**–**4**). Key insulin signalling genes, Akt1, Mtor, Irs1, Akt2, Pik3ca, Pik3r1, Foxo1, Ins1, Gsk3b, and Ins2, are directly connected with the Csf1r to mediate Ctss, C1qa, C1qc, C1qb, Ly86, and Tyrobp. These interactions are associated with complement-mediated inflammatory mechanisms, phagocytosis, and inflammation (35, 36). Further, enrichment analysis using KEGG and GO revealed pathways related to insulin resistance, including the insulin signalling pathway, longevity pathway, growth hormone activity, and neurohormone activity. These findings suggest that the PI3K/Akt signalling and insulin resistance axis may activate the complement system to promote inflammation. Expression analysis of key hub genes *Irs1, Akt1, Akt2, mTOR, Tyrobp*, and *C1qa*, along with their associated biological processes, cellular components and molecular functions, further highlights the link between diabetes and inflammation (**Figures 3A–E**).

**Table 2:** Mcode score.

Cluster	Top GO/KEGG terms	Genes in cluster	MCODE score
Cluster 1	*PI3K*-Akt Signalling, Insulin Signalling	*Akt1, Mtor, Irs1, Akt2, Pik3ca, Pik3r1, Foxo1, Ins1, Gsk3b, and Ins2*	8.5
Cluster 2	Inflammation, Phagosome, Microglial Activation	*Csf1r, Ctss, C1qa, C1qc, C1qb, Ly86, and Tyrobp*	6.6

**Table 3:** MTT assay in SHSY-5Y neuroblastoma cells.

Groups	Concentration			Replicate 1	Replicate 2	Replicate 3	Mean		IC50
		OD	%inhibition	OD	%inhibition	OD	%inhibition	%inhibition	IC50
ZnO NPs (µg/ml)	Control	0.85	0.00	0.84	0.00	0.84	0.00	0.00	**30.74**
	5	0.70	16.92	0.72	14.05	0.71	14.80	15.26	
	10	0.59	30.46	0.58	30.61	0.57	31.85	30.97	
	20	0.48	42.64	0.49	41.82	0.49	41.72	42.06	
	40	0.37	56.21	0.38	54.52	0.38	55.10	55.28	
	80	0.28	67.34	0.27	68.21	0.28	66.44	67.33	
	160	0.18	78.70	0.19	77.38	0.19	76.85	77.64	
	320	0.09	89.75	0.09	89.32	0.09	88.93	89.33	
Doxorubicin(µM)	1.56	0.773	8.52	0.773	8.00	0.774	7.66	8.06	**13.66**
	3.13	0.648	23.34	0.646	23.08	0.643	23.29	23.24	
	6.25	0.588	30.46	0.587	30.12	0.589	29.77	30.12	
	12.50	0.483	42.79	0.493	41.27	0.495	40.89	41.65	
	25.00	0.368	56.45	0.351	58.21	0.349	58.33	57.66	
	50.00	0.297	64.85	0.287	65.88	0.28	66.63	65.79	
	100.00	0.173	79.53	0.177	78.98	0.179	78.69	79.06	

**Table 4:** ROS generation assay in SH-SY5Y neuroblastoma cells.

Groups	Concentration				Replicate 1		Replicate 2		Replicate 3		Mean			±SD
		RFU (Ex:Em=434/595nm)	% ROS generation	RFU (Ex: Em=434/595nm)		% ROS generation		RFU (Ex: Em=434/595nm)		% ROS generation		% ROS generation		
Control	0.0	5428729	0.00	5688802		0.00		5895537		0.00		0.00	0.00	
ZnO NPs (µg/ml)	0	5601770	3.19	5770652		1.44		5981062		1.45		2.03	1.01	
	5	5882050	8.35	5952442		4.63		6286551		6.63		6.54	1.86	
	10	6219640	14.57	6378579		12.13		6637217		12.58		13.09	1.30	
	20	6258258	15.28	6410090		12.68		6981004		18.41		15.46	2.87	
	40	7098619	30.76	7287366		28.10		7369643		25.00		27.95	2.88	
	80	8075247	48.75	8146760		43.21		8379304		42.13		44.70	3.55	
	160	8218651	51.39	8367174		47.08		8431281		43.01		47.16	4.19	
Standard (Doxorubicin) (µM)	320	6288742	15.84	6342988		11.50		6594823		11.86		13.07	2.41	
	1.56	6811806	25.48	7177028		26.16		7225575		22.56		24.73	1.91	
	3.13	7134344	31.42	7448160		30.93		7861118		33.34		31.90	1.28	
	6.25	7906744	45.65	8147770		43.22		8154977		38.32		42.40	3.73	
	12.5	8818988	62.45	9065351		59.35		9178098		55.68		59.16	3.39	
	25	9741588	79.45	10177981		78.91		10454481		77.33		78.56	1.10	
	50	10222064	88.30	10659296		87.37		10894112		84.79		86.82	1.82	
	100													

RFU-relative fluorescence units.

### 3.2 Characterisation of green-synthesised ZnO nanoparticles

**Figure 4:**
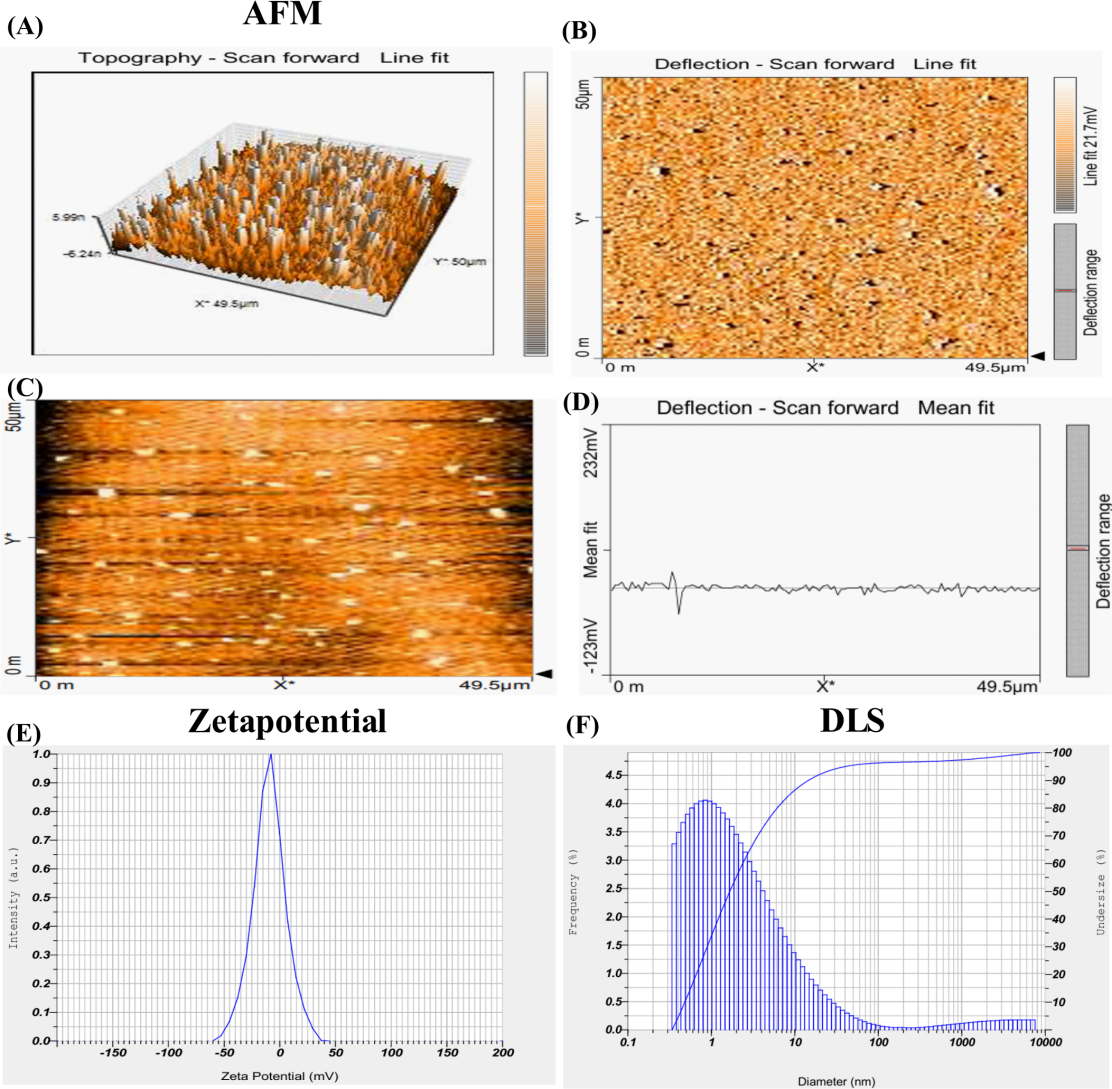
Green synthesised ZnO nanoparticles analysed in atomic force microscopy **(A, C)** Topography- scan forward, **(B, D)** Deflection – Scan forward, **(E)** Zeta potential measures charge as -24.0 mV, and **(F)** Size of the nanoparticle measured in Dynamic Light Scattering as 13.6 nm.

Our previous study detailed the green synthesis of *S. grandiflora*-mediated ZnO NPs. Leveraging that established framework, the current investigation focuses on additional characterisations to further elucidate the therapeutic properties of ZnO NPs. To gain deeper insights into the structural and functional attributes of the synthesised nanoparticles, AFM, Zeta potential, and DLS were performed. AFM results revealed the surface topography of the ZnO NPs formed through the green synthesis (**Figure 4A**). Particles were measured at 50 μm. It showed the height and dispersed particles, which are spherical and agglomerated (37). DLS measured the average diameter to be 13.6nm (**Figure 4B**). The potential charge of ZnO NPs in zeta potential is -24.0 mV with the electrophoretic mobility mean of -0.000186 cm2/Vs (**Figure 4C**). NPS activity depends on the source, shape, dose, size, charge, surface area, and electric and magnetic properties (38). The lower the charge of the NPs, the more stable; thus, ZnO NPs were considered stable (39).

### 3.3 Green-synthesised ZnO NPs reduce oxidative stress and are neuroprotective in the SH-SY5Y cell line

**Figure 5:**
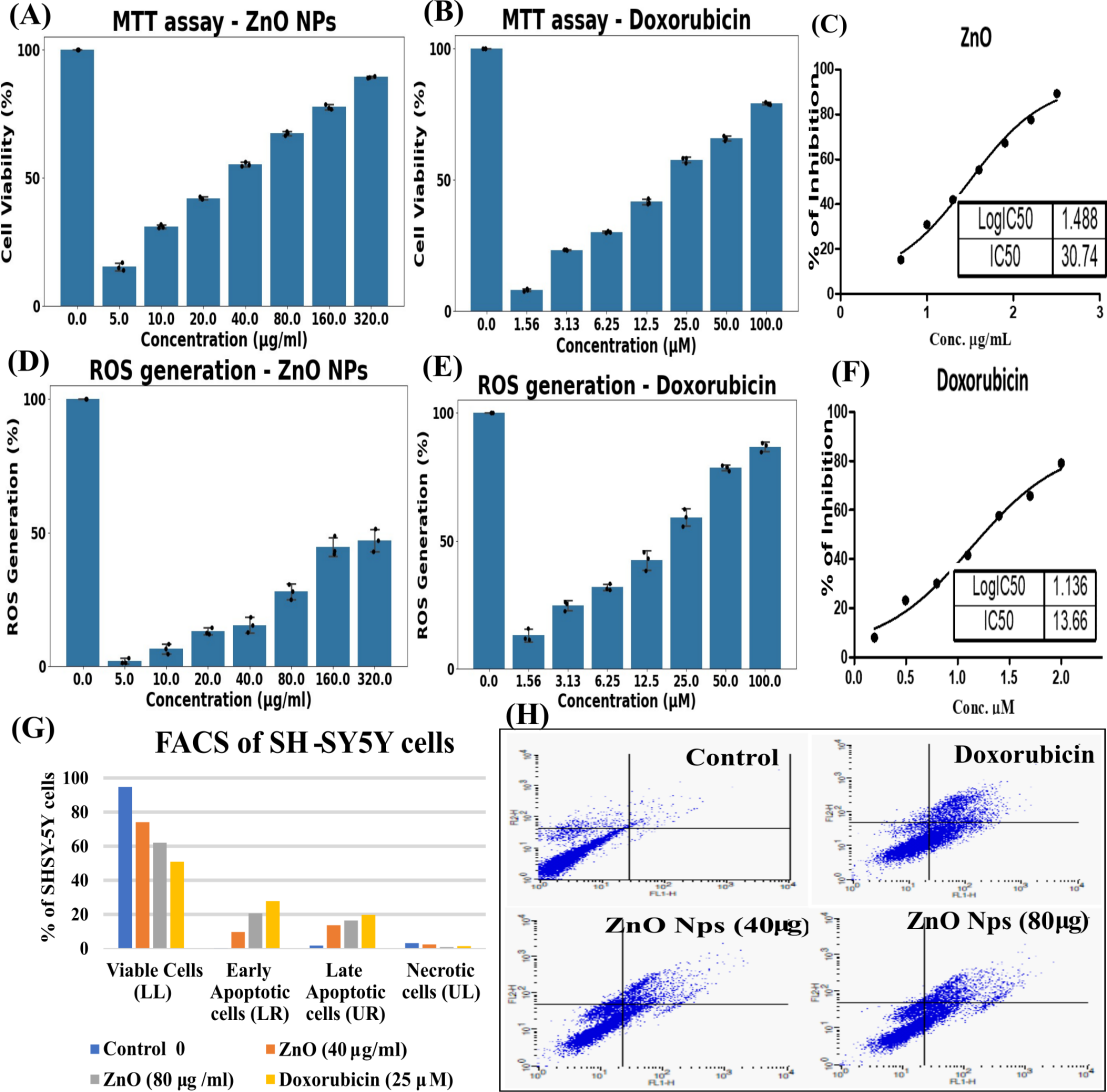
*In-vitro* cytotoxicity, apoptotic effects, and flow cytometry analysis of ZnO NPs and DOX in SH-SY5Y cells. MTT assay for **(A)** ZnO NPs (IC_50_ = 30.74µg) and **(B)** Doxorubicin (IC_50_ = 13.66µM). ROS generation for **(C)** ZnO NPs and **(D)** Doxorubicin. **(E, F)** Annexin V-FITC/PI flow cytometry showing cell viability/apoptosis distribution **(F)** Doxorubicin (25 µM) and ZnO NPs (40 and 80 µg). For **(A–D)**, n=3; data analysed via one-way ANOVA (MTT: ZnO (F = 2577.79, p<0.001), DOX (F = 3894.86, p<0.001) with Tukey’s HSD *post-hoc* test. All pairwise comparisons were significant (p<0.05). ROS: doxorubicin (F = 467.39, p<0.001), all pairs were statistically significant, while ZnO (p<0.05), all pairs were significant except 5–10, 10–20, 20–40, and 160–320 µg (n.s). **(G)** Bar graph representation of Fluorescence-Activated Cell Sorting assay, **(H)** Fluorescence-Activated Cell Sorting assay.

ZnO NPs inhibited SH-SY5Y cell proliferation with increasing concentration. ZnO NPs (320 μg/ml) exhibited 89.3% inhibition with an IC_50_ of 30.74 μg/ml. Doxorubicin (100 μM) induced 79.06% of cell death with an IC_50_ of 13.66 μM (**Figures 5A, B**) (**Table 2**). These results indicate that the ZnO NPs are less toxic than doxorubicin. Doxorubicin promotes apoptosis by inhibiting topoisomerase I and II, and generating ROS, leading to caspase activation, making it a suitable standard for cytotoxic and ROS assays (40). In comparison, ZnO NPs (360 μg/ml) induced 43% of ROS generation, whereas doxorubicin (100 μM) generated 84.7% (**Figures 5C, D**) (**Table 3**). This suggests that ZnO NPs may generate fewer hydroxyl and peroxyl radicals than doxorubicin. Furthermore, ZnO NPs may regulate the antioxidant system through malondialdehyde, superoxide dismutase, and glutathione peroxidase (12). Free radicals promote AGE formation, oxidation of proteins, lipids, and nucleic acids, mitochondrial dysfunction, inflammatory cytokine release, proteasome dysfunction, protein aggregation, and apoptosis (14, 41, 42). Cells constantly produce various types of ROS through metabolic and cellular activities. However, antioxidant systems, antioxidant molecules, and certain medicines prevent this by converting ROS into less reactive species (43).

Biologically synthesised ZnO NPs have been shown to reduce free radicals at low doses, thereby attenuating ROS-mediated mitochondrial and ER dysfunction, lipid peroxidation, inflammation, DNA damage, and premature ageing (42, 44). In contrast, high accumulation of ZnO NPs and Zn^2+^ ions may promote autophagy and lysosomal-mediated cell death (**Table 4**). Zn^2+^ ions alter intracellular signalling over time in PC1 cell lines (41). The Annexin V-FITC/PI apoptosis assay indicated ZnO NPs (40 µg/ml) induced fewer cells in early and late apoptotic stages. In contrast, doxorubicin (25 μM) promoted apoptosis (early and late) as evidenced by an increased proportion of cells in the upper right quadrants.

ZnO NPs (80 µg/ml) induced early and late apoptosis more than 40 µg/ml, as shown in the increase of apoptotic cells in upper and lower quadrants (**Figures 5E, F**) (**Table 5**). ZnO NPs may modulate apoptotic gene expression (*bcl2* and caspases), oxidative stress, AGEs, and mitochondrial, ER, and Golgi dysfunctions (45). ZnO NPs influence major signalling pathways, including PI3K/Akt/mTOR, ERK, MAP/MAPK, RAS/RAF, and NF-kB, thereby regulating cell growth, survival, and apoptosis in various diseases, such as cancer, neurodegeneration, diabetes, pancreatitis, NAFLD, and hepatitis (12, 13, 44, 46). At therapeutic doses, ZnO NPs may alleviate neurodegeneration, enhance antioxidant mechanisms, and cell survival and metabolic pathways (14, 41).

**Table 5:** Calculated apoptotic cell percentage.

S. No.	Sample details	Conc. µg/ml	Viable cells (LL)	Early apoptotic cells (LR)	Late apoptotic cells (UR)	Necrotic cells (UL)
1	Control	–	94.77	0.26	1.73	3.24
2	Doxorubicin	25 µM	50.91	27.82	19.83	1.44
3	ZnO NPs	40	74.1	9.77	13.65	2.48
4		80	62.11	20.64	16.42	0.83

### 3.4 ZnO NPs alleviate hyperglycaemia and hyperinsulinemia in the D-galactose-ingested animal model

**Figure 6:**
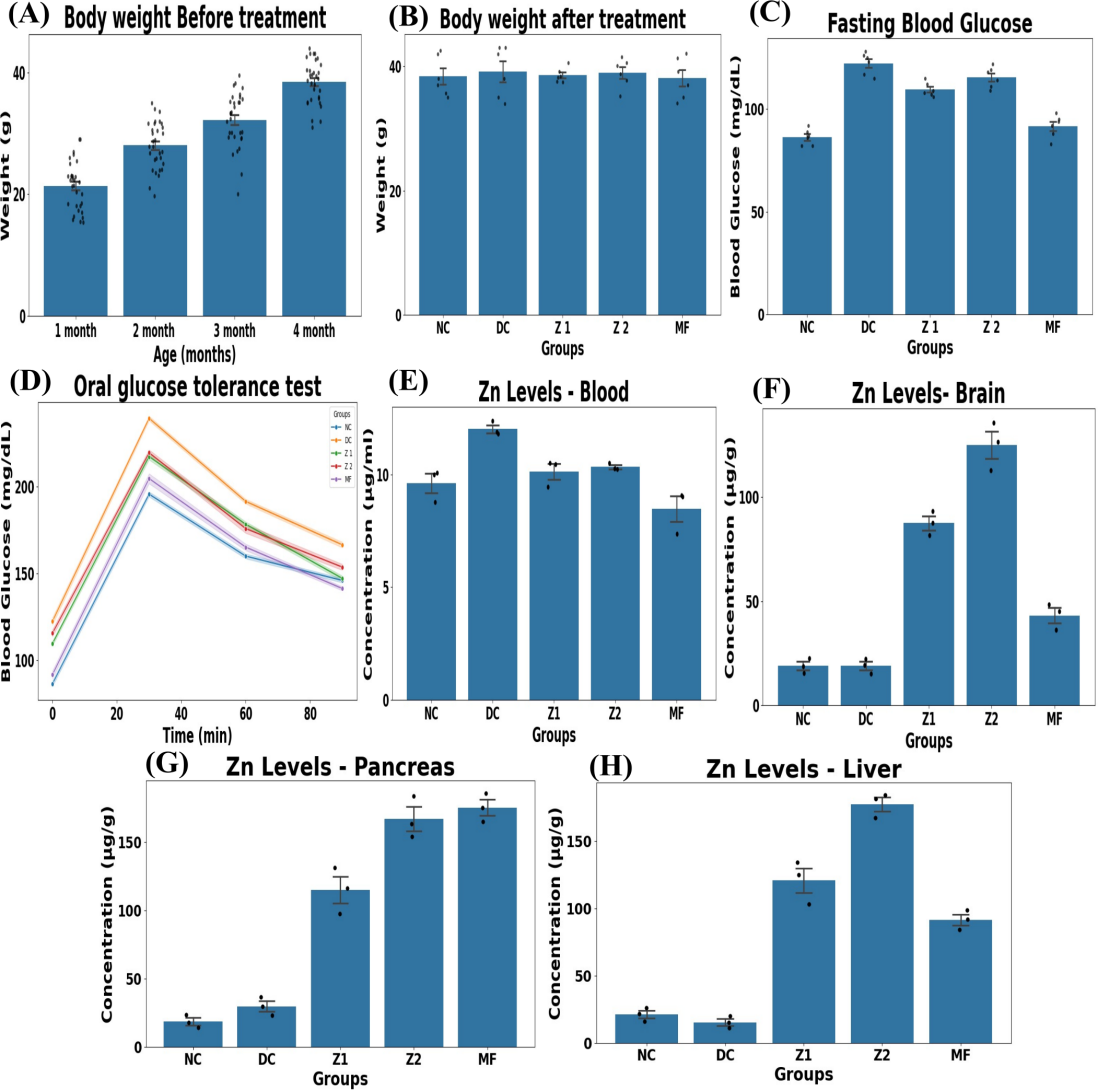
Assessment of body weight, glycaemic levels and biodistribution of Zn in mice. **(A)** Body weight change over time (n=30), weight increased significantly with age (p<0.001, and all monthly pairwise comparisons were significant. **(B)** Average body weight across groups (n=6) showed no significant differences, one-ANOVA (F = 0.127 and p=0.971) and Tukey’s HSD (P>0.05). **(C)** Fasting blood glucose level (n=6). (F = 66.35, p<0.001) and revealed DC higher than NC (p<0.05), MF and Z1 were lower than DC (p>0.05), Z2 did not differ significantly from DC (p=0.113), and Z1 and Z2 (p=0.200) had no significant difference. **(D)** Oral glucose tolerance test (n=6), significant differences occurred between groups at all time points (p<0.001). Tukey’s HSD showed DC higher than NC (p>0.05), Z1 and Z2 were significantly lower than DC (p>0.05), Z1 lower than Z2 (p=0.029). Zn levels (n=3) in **(E)** Blood, significant variance observed between groups (F = 12.59, and p< 0.001). *Post hoc* analysis showed a significant increase in Zn levels in DC (p < 0.05) compared with ZC, Z1, and Z2 (no significant difference). **(F)** Brain, highly significant increase in Zn levels observed (F = 140.48 and p<0.001). Z1 and Z2 showed higher Zn levels than NC, DC and MF (p<0.001). **(G)** Pancreas- highly significant across the groups (F = 117.56, p<0.001) and increasing Zn in Z1 and Z2. **(H)** Liver- massive Zn uptake (F = 163.89, P<0.001). Z1 and Z2 had elevated Zn levels compared to other groups (p<0.05). MF- no significant increase compared to NC and DC (p<0.05).

Body weight was gradually increased for 4 months before the induction of hyperglycaemia, IR and ageing with D-galactose. Neither D-galactose nor the ZnO NPs significantly affected body weight (**Figures 6A, B**), indicating that C57BL/6 mice did not develop obesity under these conditions. D-galactose ingestion mimics ageing by increasing ROS, AGEs, neurodegeneration, IR, and oxidative stress, and diminishes the antioxidant system by reducing malonaldehyde, superoxide dismutase, and total antioxidant status (47, 48). However, chronic D-galactose treatment may reduce body weight (49, 50), whereas ZnO NPs may help maintain it. Blood glucose level is a major biomarker for IR and diabetes-related conditions. According to WHO criteria, a fasting blood glucose level between 100 and 125 mg/dl indicates IR and hyperglycaemia (1, 51). Compared with the DC and Z2 groups, the Z1 and MF groups showed lower FBG levels. While DC had ~122 mg/dl, whereas Z1 and MF had ~109 and ~91mg/dl, respectively (**Figure 6C**), indicating hyperglycaemia in the DC group. Prolonged hyperglycaemia and IR are associated with metabolic syndrome, obesity, DM, neurodegenerative diseases, nephropathy, neuropathy, retinopathy, and cardiovascular diseases (51, 52).

The OGTT assesses glucose tolerance by measuring blood glucose at intervals (30 to 90 min). Glucose levels decreased over time as insulin promotes glucose uptake in cells. Notably, blood glucose levels in NC, Z1, and MF were reduced at 90 min compared with Z2 and DC groups (**Figure 6D**) (53). *S. grandiflora* mediated ZnO NPs efficiently in alleviating hyperglycaemia. ICP-MS analysis further demonstrated increased Zn bioavailability in the blood, brain, liver, and pancreas in the Z1 and Z2 groups compared with non-ZnO NPs-treated groups (NC, DC, and MF). ZnO NPs clearance rate is high in the blood but moderate in the liver, and lower retention in the pancreas and brain (**Figures 6E–H**). These findings suggest that ZnO NPs may cross the BBB and enhance their potential in treating neural diseases and brain IR through Zn ion release (54). Biodistribution and bioavailability are factors that determine the NPs’ efficacy, half-life, and clearance (55).

**Figure 7:**
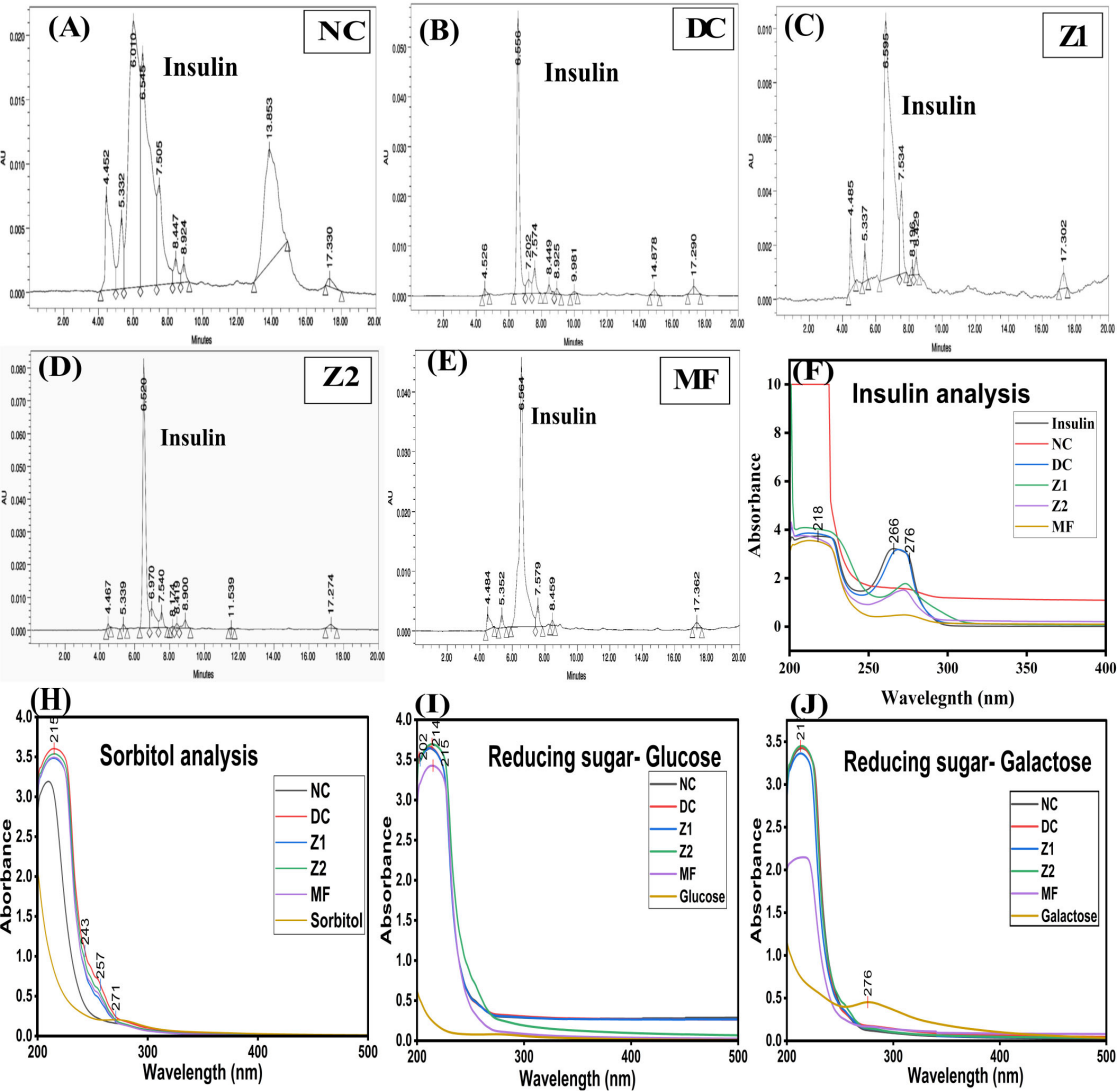
Insulin, sorbitol, and reducing sugar analysis. **(A–E)** Insulin analysis with High Performance Liquid Chromatography, UV-Vis spectroscopy analysis of **(F)** UV-Vis spectroscopy analysis of insulin, **(G)** Sorbitol, **(H)** Glucose, **(I)** Galactose, and **(J)** Glucose.

Blood insulin levels (normal range: 5 to 40 μU/L) play an important role in regulating glucose uptake; however, they may fluctuate due to hyperinsulinemia, hypoinsulinemia, pregnancy, IR, obesity, and after meals. Insulin levels are directly associated with pancreatic function, insulin biosynthesis, insulin secretion, body mass index, blood glucose levels, and ageing (56, 57). Significant differences in insulin levels NC, DC, Z1, Z2, and MF groups. DC, Z2, and MF groups showed hyperinsulinemia, with DC and MF showing moderate increases, and Z2 displayed an elevation. In contrast, Z1 has an insulin level lower than the NC (**Figures 7A–F**). These findings reveal that insulin secretion may be influenced by ZnO NPs, especially with Z1, which appears to treat the hyperinsulinemia.

The reduction in blood glucose levels was reflected in insulin levels; however, the Z2 exhibited hyperinsulinemia, suggesting a dose-dependent effect of ZnO NPs on insulin synthesis, secretion, and signalling mechanisms (58, 59). Zn^2+^ ions and ZnO NPs maintain insulin structure through stabilising disulphide bonds (28, 53, 55). Notably, green-synthesised ZnO NPs improve insulin secretion by protecting the pancreatic β cells’ mass and structure. In addition, *S. grandiflora* has been reported to alleviate diabetic complications, increase insulin and C-peptides and reduce LDL (59, 60).

### 3.5 ZnO NPs alter the sorbitol, reducing sugar, methyl glyoxal, and conjugated dienes

Sorbitol is synthesised through the polyol pathway under hyperglycaemic conditions. It accumulates in erythrocytes, eyes, kidneys, and brain, leading to diabetic complications, such as cataracts, neuropathy, and nephropathy. In the brain, sorbitol alters neurotransmitters and causes neural dysfunction (61). Sorbitol levels in erythrocytes are directly proportional to the blood glucose. UV spectral analysis at 215, 242, 256, and 271 nm showed that NC had the lowest level of sorbitol compared to DC, Z1, Z2, and MF (**Figure 7G**). Reducing sugars, glucose and galactose absorbed at 210 and 275 nm (**Figures 7H–I**), consistent with earlier blood glucose measurements.

**Figure 8:**
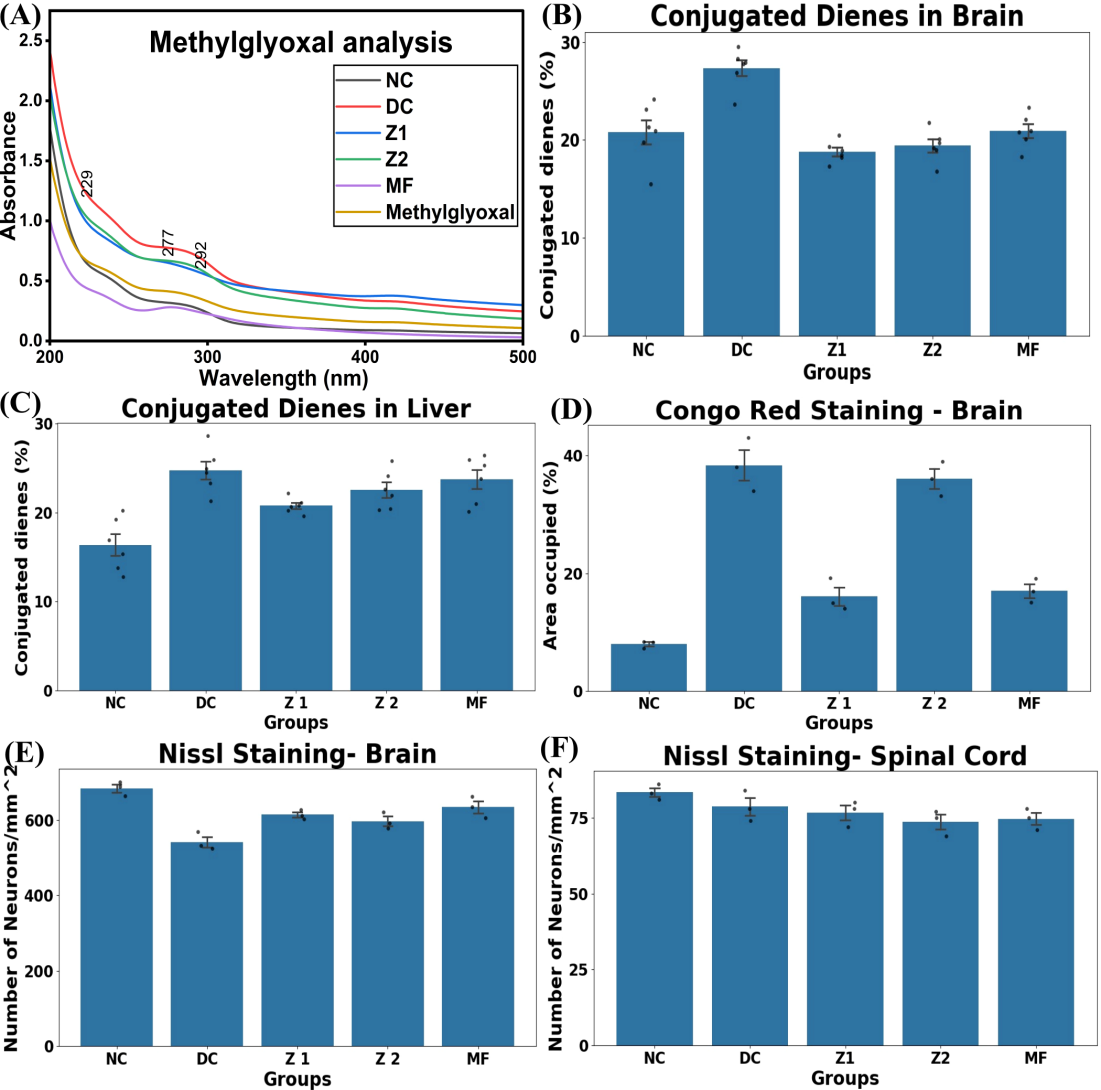
Methyl glyoxal, lipid peroxidation and insulin analysis. **(A)** UV analysis of methylglyoxal. Conjugated dienes levels (n=6) in **(B)** Brain, one-way ANOVA followed by Tukey’s HSD revealed conjugated dienes (%) elevated in DC groups compared to the NC group (p<0.001). Z1 and Z2 were significantly attenuated in the brain (p<0.001). **(C)** Liver, a significant elevation in conjugated dienes was observed in DC compared to NC (p<0.001), Z1 reduced conjugated dienes compared to Z1 and DC (p<0.05). Histopathological analysis **(D)** amyloid beta occupied area in brain (n=3), one-way ANOVA followed by Tukey’s HSD showed a significant increase in the affected area was observed in DC than in NC (p<0.001), Z1 and MF significantly lowered the amyloid deposited area (p<0.001 vs DC), while Z2 showed a significant increase than DC (p>0.05), suggesting dose dependent threshold. Nissl staining of **(E)** Nissl staining of the Brain, a significant reduction in neural density observed in DC compared to NC (p< 0.001), Z1 and MF significantly mitigated the loss of neural loss (p<0.05 and p<0.01 vs DC, respectively). Z2 did not show a statistically significant (p>0.05) effect. **(F)** Nissl staining of spinal cord No significant differences observed between groups (p>0.05).

Methylglyoxal (MG) is a highly reactive intermediate from metabolic processes, e.g., glycolysis. It glycates lipids, nucleic acids, and proteins by binding to arginine, lysine, and cysteine residues (62). Under normal circumstances, MG metabolises into lactic acid; however, in IR, its accumulation causes cellular dysfunction, tissue damage, and diabetic complications (48). UV spectra (200–300 nm) disclosed that DC exhibited elevated levels of MG than NC, followed by Z2, Z1, and MF. This indicated that metformin and ZnO NPs significantly enhanced the MG clearance (**Figure 8A**). D-galactose may elevate the MG accumulation by impairing the glyoxalase system, thereby reducing MG detoxification ability and antioxidant mechanisms, ultimately by promoting inflammation via activating NF-κB, RAGE, TNFα, ILs, and ERK-mediated signalling pathways (47, 48). ALPs may induce genotoxicity, proteotoxicity, cellular senescence, and oxidative stress; activate inflammatory cytokines and interleukins; and promote obesity, ageing, and age-related diseases (63).

Conjugated dienes are a kind of ALPs formed through lipid peroxidation, acting as an indicator for oxidative stress and inflammation, and affect protein structure and accumulation (64, 65). In this study, conjugated dienes were found to be higher in the brain and liver of DC than in NC, Z1, Z2, and MF. ZnO NPs and metformin efficiently reduced these levels, with Z1 nearing the NC range (**Figures 8B, C**). This supports that lipid peroxides are unstable in the presence of heavy metals like Zn and Fe by the Fenton reaction (66, 67). ZnO NPs facilitate ALPs (conjugated dienes) and methylglyoxal clearance and act as antioxidants or pro-antioxidants to scavenge and inhibit the free radicals.

### 3.6 ZnO NPs act on D-galactose-induced liver damage and amyloid-β clearance in the brain

**Figure 9:**
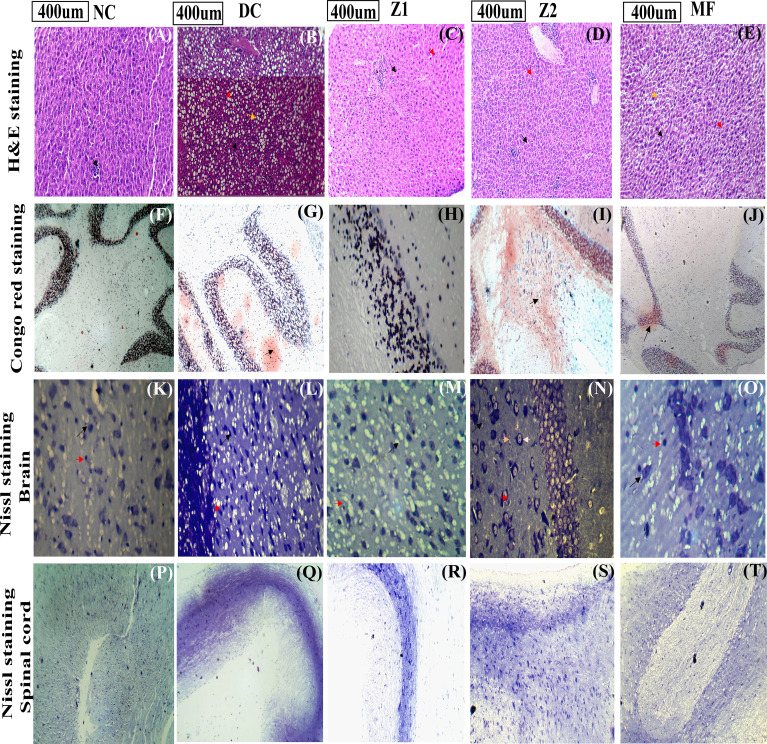
Histopathological analysis **(A–E)** H&E staining of the liver. The black arrow - inflammation, the yellow arrow- fatty liver, and the red arrow - sinusoidal structure. **(F–J)** Congo red staining of the brain, the black arrow - amyloid-b deposition. **(K–O)** Nissl staining of the brain, the black arrow - neurons, red arrow- astrocytes, yellow- neuron membrane, White- endoplasmic reticulum and ribosomes. **(P–T)** Nissl stain of the Spinal cord, the black arrow - motor neurons, and the red arrow - astrocytes.

H&E staining outlines cell structure in detail and identifies pathological abnormalities (68). In this study, liver tissues of NC, DC, Z1, Z2, and MF were analysed using H&E staining (**Figures 9A–E**). The NC showed intact hepatocytes, clear sinusoidal patterns, and no inflammation. In contrast, the DC group exhibited fat deposition, liver injury, inflammation, necrosis, apoptosis, and congestion, indicating features of NAFLD and associated inflammation. The presence of neutrophils, lymphocytes, and eosinophils confirms ongoing inflammation. These findings support that D-galactose induces oxidative stress, AGEs, inflammation, and cellular senescence, contributes to ageing, IR, and NAFLD development (50, 69, 70). Instead, the Z1 group showed healthy hepatocytes with minimal fat deposition, minimal aggregations of neutrophils, lymphocytes, and eosinophils, signifying mild inflammation and a reversal of NAFLD. The MF group also showed a reduction in inflammation and fat deposition. However, Z2 exhibited increased inflammation, apoptosis, and fat accumulation compared to the NC, DC, and Z1 groups.

Fibrotic changes were observed in DC, Z1, Z2, and MF groups, indicating chronic liver injury and scarring. ZnO NPs have been reported to improve inflammation, mitochondrial inflammation, NAFLD, hepatic steatosis, peripheral insulin resistance, and dysregulated hepatic lipogenesis (44, 71). Furthermore, inflammation and lipid accumulation in the liver may contribute to brain metabolic dysfunction via the liver-brain axis. Lipid compounds such as ceramide and palmitate, along with apolipoproteins and lipid transporters, can promote insulin resistance, neuroinflammation, and disrupt neural integrity (6, 72).

Congo red staining detects the amyloid-β deposition, a hallmark of neurodegenerative diseases and brain IR. Amyloid-β triggers mitochondrial dysfunction, protein ubiquitination, oxidative stress, inflammation, astrocyte accumulation, and Tau hyperphosphorylation (2, 12, 73). Amyloid-β deposition was observed in DC, Z1, Z2, and MF groups. Z1 had minimal amyloid-β presence, suggesting enhanced clearance and a prominent. In contrast, Z2 amyloid-β accumulation is comparable to DC, indicating a potential aggravating effect and depicting a dose, size, surface area, and shape-dependent effect of ZnO NPs. Metformin also demonstrated a reduction in amyloid-β accumulation (**Figures 8C**, **9F–J**). These verdicts support a dose-dependent bidirectional relationship of Zn, whereas it may promote amyloid-β accumulation, neuroinflammation, and neuroapoptosis, while modulating PI3K/Akt, ERK, and inflammatory signalling pathways (54, 74–76).

Conversely, Zn also reported to inhibit amyloid-β synthesis, improve enzymatic and non-enzymatic clearance, and regulate antioxidant enzyme activities (12, 56). Amyloid-β preferentially accumulates in regions of the amygdala, cerebellum, and hypothalamus; for instance, cerebellum deposition is associated with motor impairments affecting coordination, posture, balance, and motor learning (77, 78). *S. grandiflora* and its ZnO NPs possess significant neuroprotective, antioxidant, hyperglycaemic, and hyperinsulinemic properties, which are suitable for treating IR and neurodegeneration (60, 79, 80). Meanwhile, Zn supplementation has been shown to improve cognitive function and oxidative stress, contributing to brain protection (54, 79).

Nissl staining, which highlights RNA, proteins, and rough endoplasmic reticulum, provides a structural outline of neurons and helps to assess neuronal integrity (81). It revealed no significant difference in neuronal density (per cm^2^) among the NC, Z1, Z2, and MF groups, but the DC group showed a slight reduction (**Figures 8E**, **9K–O**). Astrocyte presence indicated neuroinflammation with the highest in DC, moderate in Z2, and the lowest in Z1. Neuron morphology appeared irregular in the DC and MF groups, pointing to structural damage, while Z2 showed minimal structural alteration. This suggests that amyloid-β accumulation alone may not directly cause neurodegeneration, as the amyloid-β-affected area was high in Z2 (82). However, chronic neuroinflammation can induce apoptosis through disrupting PI3K/Akt signalling, IR, AGE, ROS generation, and oxidative stress (83). Nissl staining of the spinal cord further showed that NC has a normal distribution of Nissl bodies and healthy neurons. However, D-galactose treatment resulted in mild loss of neuronal density, fragmentation, shrinkage, and neuronal damage, but it did not cause severe noticeable damage in neuronal integrity (**Figures 8F**, **9P–T**). This suggests that the lower dose of ZnO NPs promoted neural integrity and structure. While previous experiments discussed neurotoxicity, hepatotoxicity, and pancreatic β islets destruction (45, 46), this study suggests that ZnO NPs possess neuroprotective activity in a dose-dependent manner. Amyloid-β accumulation, Tau hyperphosphorylation, oxidative stress, IR, AGEs, and ALPs induce neurodegeneration, which can be slowed down but not reversed. ZnO NPs and Zn supplementation improve motor neuron disease symptoms and attenuate sensory and locomotor functions in PD, AD, and ischemia (14, 46, 56).

### 3.7 *pi3k-ager-app* correlation can be altered with ZnO NPs to stimulate insulin sensitivity and amyloid-β clearance

**Figure 10:**
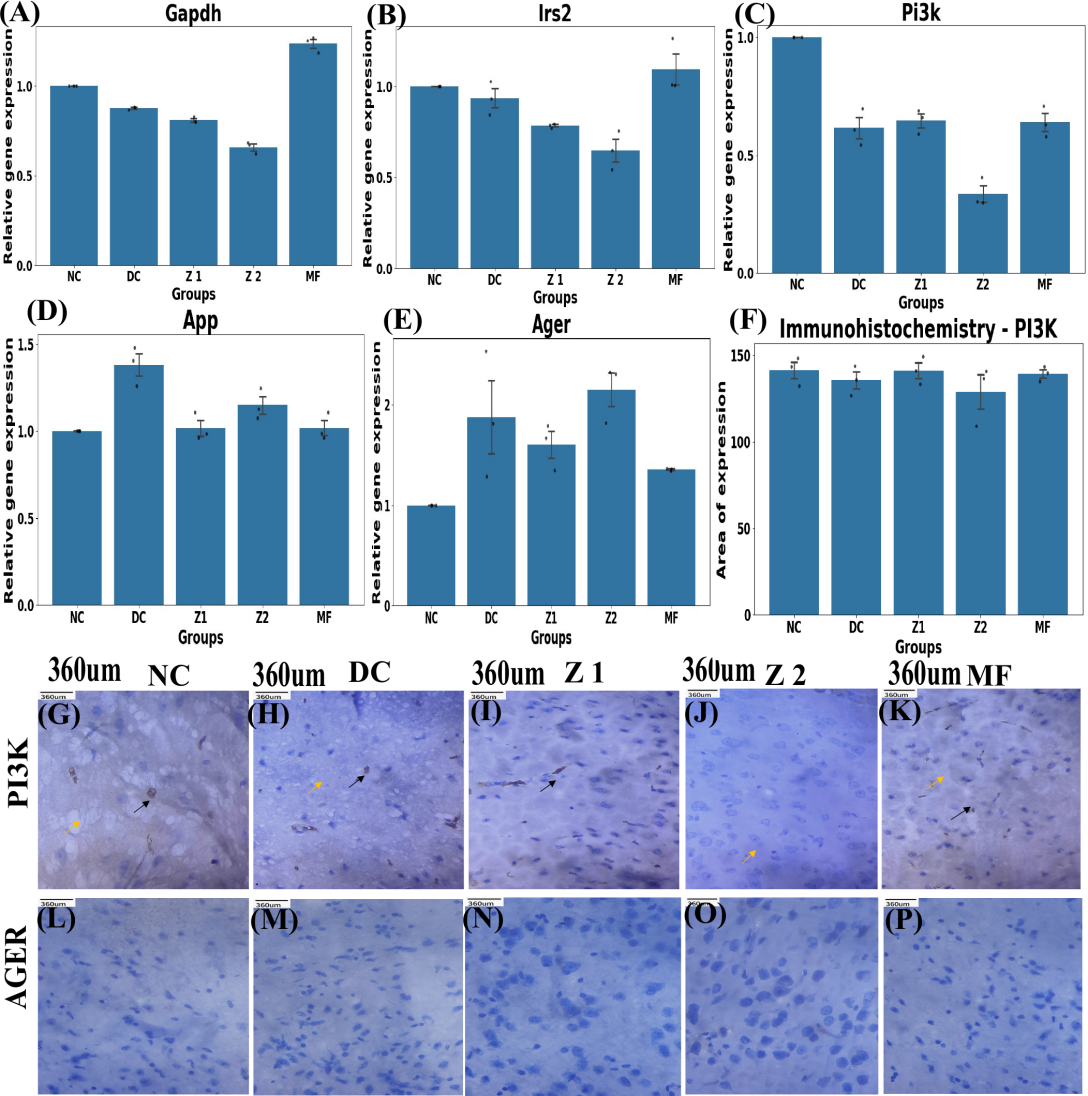
Relative gene expressions (n=3) of **(A)** *Gapdh* showed significant fluctuation (p<0.001) across the groups, signifying MFs’ role in elevating glucose metabolism compared to all other groups. **(B)** *App*, significant upregulation observed in DC (p<0.001 vs NC), *app* expression restored in Z1 and MF (p< 0.01 vs DC), while Z2 (p< 0.05) reduced expression of *app*, but less than Z1. **(C)** *Ager*, significant upregulation observed in DC compared to NC (p<0.05 vs DC), Z1 and MF (p>0.05 vs DC) significantly reduced *Ager* levels compared to DC, Z2 (p=0.01 vs NC) exhibited elevated *Ager* levels than NC. **(D)** irs 2, significant difference among the groups was observed (p< 0.01), Z1 AND MF were comparable to NC, Z2 downregulation relative to NC, DC, and MF (p<0.05). **(E)** *pi3k*, DC exhibited marked upregulation, relative to NC (p<0.01). Z1, Z2 and MF significantly attenuated this upregulation (p<0.05 for Z2, p<0.01 for Z1 and MF). Significance was analysed with one-way ANOVA and Tukey’s *post-hoc* test. **(F)** Area of *PI3K* expression in the Brain revealed the statistical difference across groups as p>0.05. **(G–K)** Expression of *PI3K* protein, black arrow - nucleus of occasional epithelial cells, and Yellow - neurofibrillary matrix. **(L–P)** Expression of *AGER* protein.

D-galactose-induced ageing, brain IR, and neurodegeneration through influencing key genes, including *Irs 2, pi3k, ager*, and *app*. In the NC group, *pi3k* and *Irs-2* expression levels were high, indicating normal cellular function, but low in the DC and MF groups, moderate in the Z1 and Z2 groups. This reduction implies the risk of developing brain IR. Markedly, ZnO NPs treatment efficiently improved *pi3k* and *irs-2* expressions. *Ager* expression is typically low under physical conditions but increases with the presence of AGE (84). Consistently in NC, *ager* expression was low but amplified in DC, which seems to be reduced following ZnO NPs and metformin ingestion. In Z1 and MF groups, *ager* expressions were close to NC, reflecting decreased AGE accumulation. AGE reduction likely to influence *app* expression, therefore attenuating oxidative stress, neuroinflammation, and brain IR (85). This decline may have influenced *app* expression, and it was dysregulated after D-galactose treatment, but improved with ZnO NPs and metformin administration, suggesting coordinated regulation between *app* and *ager* (**Figures 10A–E**).

Immunohistochemistry of PI3K protein revealed total area of expression remained statistically consistent across all groups. However, a slight decrease was observed in DC and Z2, depicting functional impairment despite stable overall expression, affecting insulin sensitivity (**Figures 10F–N**). This suggests altered pathway activity rather than overall protein abundance. These outcomes highlight the mechanistic link between brain IR and neurodegeneration through the IRS/*PI3K* signalling pathway and amyloid-β deposition. ZnO NPs may alter the PI3K pathway, resulting in regulation of cell growth, development, and metabolism (14, 86, 87), and potentially slowing neurodegeneration (15, 17, 88). In contrast, AGER expression is low or undetectable across all groups (**Figures 10O–S**), despite variation in gene expression. This divergence indicates translation of mRNA may not correlate significantly with protein levels due to translation efficiency, protein stability, and technical variability (87, 89, 90).

## 4 Conclusion

In conclusion, brain IR promotes neurodegeneration, exacerbated by lifestyle, ageing, and environmental factors. This study demonstrates a mechanistic link between insulin signalling and inflammatory pathways through the IRS/PI3K/RAGE/APP axis. ZnO NPs showed dose-dependent activity with Z1 dose reducing ROS generation, hyperglycaemia, hyperinsulinemia, and glycation products, while improving neural integrity. Importantly, ZnO NPs crossed the BBB, facilitating amyloid-β deposits clearance, maintaining the shape and structure of neurons in the brain and spinal cord, and regulating expression of the key genes in IRS/PI3K/RAGE/APP signalling pathway. These findings emphasise the therapeutic potential of ZnO NPs in managing metabolic and neurodegenerative diseases. However, clinical translation of the ZnO NPs needs further investigation into long-term safety, optimal dosing, and pharmacokinetics. Overall, ZnO NPs represent a promising strategy for targeting metabolic disorders and their related pathological outcomes.

## Data Availability

The original contributions presented in the study are included in the article/**Supplementary Material**. Further inquiries can be directed to the corresponding author.
