# Correction: Zinc oxide nanoparticles mitigate insulin resistance in a D-galactose-induced C57BL/6 mouse model

**DOI:** 10.3389/fendo.2026.1879196

**Published:** 2026-05-22

**Authors:** Kanagavalli Ramasubbu, Devi Rajeswari V.

**Affiliations:** Department of Biomedical Sciences, School of Biosciences and Technology, Vellore Institute of Technology, Vellore, Tamil Nadu, India

**Keywords:** amyloid-β deposition, insulin resistance, insulin signalling, neurodegeneration, ZnO nanoparticles

There was a mistake in **Figure 9** as published. The corrected **Figure 9** includes panels **(A–E)** showing H&E staining, along with revised labelling of panels **(F–T)**, which were not updated in the originally published version. The corrected **Figure 9** appears below.

**Figure 9:**
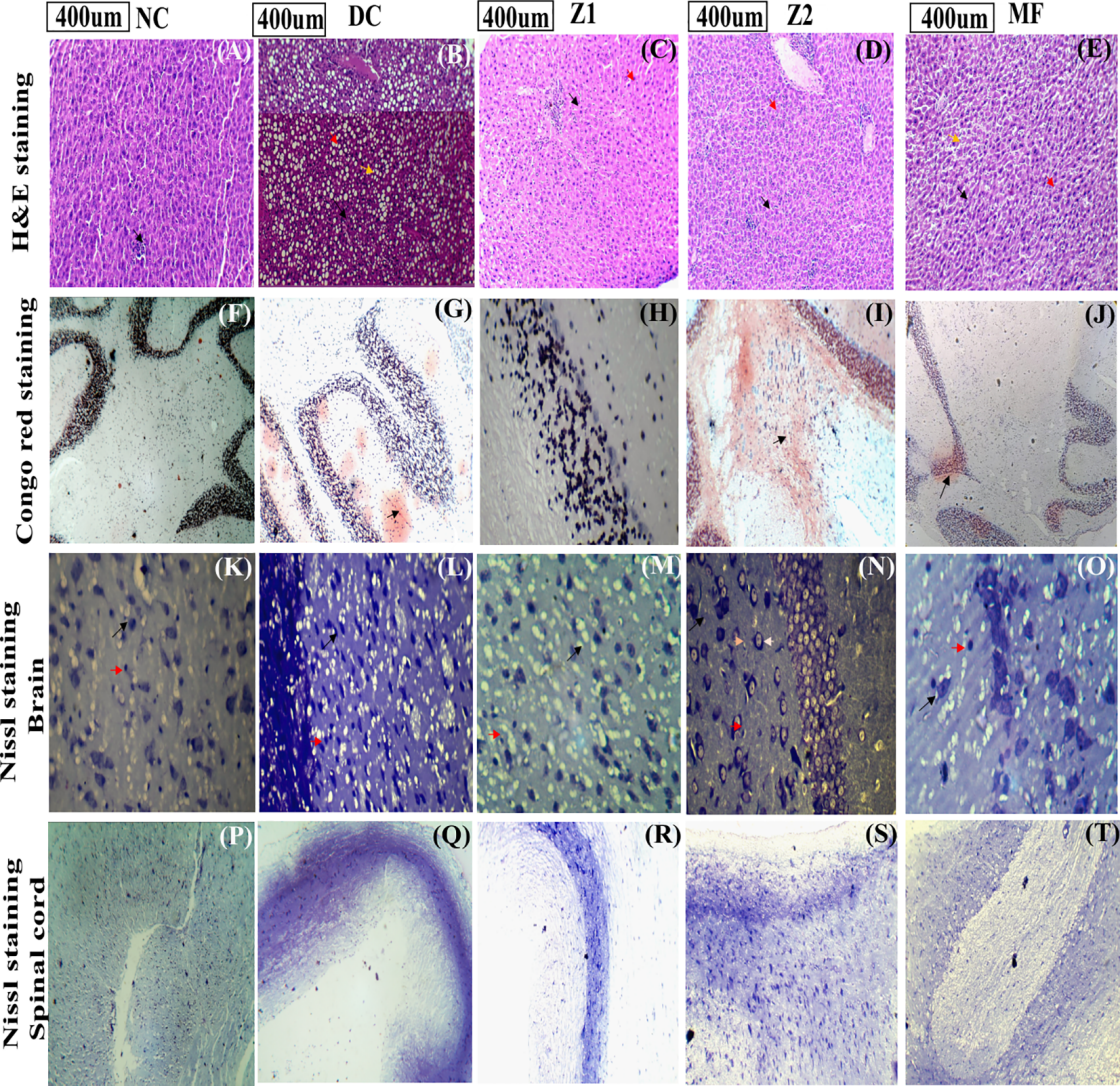
Histopathological analysis **(A–E)** H&E staining of the liver. The black arrow - inflammation, the yellow arrow- fatty liver, and the red arrow - sinusoidal structure. **(F–J)** Congo red staining of the brain, the black arrow - amyloid-β deposition. **(K–O)** Nissl staining of the brain, the black arrow - neurons, red arrow- astrocytes, yellow- neuron membrane, White- endoplasmic reticulum and ribosomes. **(P–T)** Nissl stain of the Spinal cord, the black arrow - motor neurons, and the red arrow - astrocytes.

The original version of this article has been updated.

