# Supplementary material for: Zinc oxide nanoparticles mitigate insulin resistance in a D-galactose-induced C57BL/6 mouse model
Source: Front Endocrinol (Lausanne). 2026 May 12;17:1819985. doi: 10.3389/fendo.2026.1819985 (PMC13237717; doi:10.3389/fendo.2026.1819985)
Supplement: Supplementary file 1 [file Table1.docx]

| **Index** | **Name** | **P-value** | **Adjusted p-value** | **Odds Ratio** | **Combined score** |
| --- | --- | --- | --- | --- | --- |
| 1 | Insulin resistance | 7.092e-16 | 5.814e-14 | 152.15 | 5307.38 |
| 2 | Longevity regulating pathway - multiple species | 7.964e-16 | 5.814e-14 | 235.82 | 8198.71 |
| 3 | Adipocytokine signaling pathway | 2.921e-15 | 1.422e-13 | 197.69 | 6615.94 |
| 4 | Insulin signaling pathway | 6.972e-15 | 2.545e-13 | 116.31 | 3791.49 |
| 5 | Longevity regulating pathway | 1.791e-14 | 5.229e-13 | 155.01 | 4906.77 |
| 6 | Regulation of lipolysis in adipocytes | 1.335e-13 | 3.249e-12 | 195.37 | 5791.74 |
| 7 | Growth hormone synthesis, secretion and action | 2.202e-13 | 4.593e-12 | 111.16 | 3239.54 |
| 8 | Ampk signaling pathway | 2.704e-13 | 4.934e-12 | 108.19 | 3130.85 |
| 9 | Prostate cancer | 8.559e-12 | 1.250e-10 | 103.56 | 2639.06 |
| 10 | Chagas disease | 8.559e-12 | 1.250e-10 | 103.56 | 2639.06 |

**Supplementary Table 1 KEGG analysis**

**Supplementary Table 2: GO Biological process analysis**

| **Index** | **Name** | **P-value** | **Adjusted p-value** | **Odds Ratio** | **Combined score** |
| --- | --- | --- | --- | --- | --- |
| 1 | Cellular Response to Insulin Stimulus (GO:0032869) | 7.732e-16 | 4.856e-13 | 150.61 | 5240.48 |
| 2 | Response to Insulin (GO:0032868) | 1.095e-14 | 3.440e-12 | 165.53 | 5320.98 |
| 3 | Cellular Response to Peptide Hormone Stimulus (GO:0071375) | 3.397e-14 | 7.112e-12 | 142.35 | 4414.63 |
| 4 | Insulin Receptor Signaling Pathway (GO:0008286) | 5.163e-14 | 8.106e-12 | 226.53 | 6930.75 |
| 5 | Positive Regulation of Glucose Metabolic Process (GO:0010907) | 6.483e-12 | 7.943e-10 | 479.95 | 12364.45 |
| 6 | Cell Surface Receptor Protein Tyrosine Kinase Signaling Pathway (GO:0007169) | 7.589e-12 | 7.943e-10 | 51.46 | 1317.60 |
| 7 | Positive Regulation of D-glucose Import (GO:0046326) | 4.001e-11 | 3.590e-9 | 311.86 | 7466.47 |
| 8 | Positive Regulation of D-glucose Transmembrane Transport (GO:0010828) | 1.275e-10 | 1.001e-8 | 239.82 | 5463.87 |
| 9 | Regulation of D-glucose Import (GO:0046324) | 2.819e-10 | 1.967e-8 | 201.09 | 4421.86 |
| 10 | Regulation of Fatty Acid Beta-Oxidation (GO:0031998) | 6.380e-10 | 4.007e-8 | 522.09 | 11054.06 |

**Supplementary Table 3: GO Cellular component analysis**

| **Index** | **Name** | **P-value** | **Adjusted p-value** | **Odds Ratio** | **Combined score** |
| --- | --- | --- | --- | --- | --- |
| 1 | Phosphatidylinositol 3-Kinase Complex, Class I (GO:0097651) | 0.00002929 | 0.0007176 | 350.40 | 3657.62 |
| 2 | Phosphatidylinositol 3-Kinase Complex, Class IA (GO:0005943) | 0.00002929 | 0.0007176 | 350.40 | 3657.62 |
| 3 | Protein Kinase Complex (GO:1902911) | 0.0002395 | 0.003912 | 105.05 | 875.77 |
| 4 | Collagen-Containing Extracellular Matrix (GO:0062023) | 0.0006120 | 0.007498 | 12.17 | 90.03 |
| 5 | Phagocytic Vesicle (GO:0045335) | 0.004885 | 0.03668 | 21.35 | 113.64 |
| 6 | Neuronal Dense Core Vesicle (GO:0098992) | 0.005239 | 0.03668 | 249.69 | 1311.24 |
| 7 | TORC1 Complex (GO:0031931) | 0.005239 | 0.03668 | 249.69 | 1311.24 |
| 8 | TORC2 Complex (GO:0031932) | 0.007328 | 0.04488 | 166.44 | 818.24 |
| 9 | Endocytic Vesicle (GO:0030139) | 0.01862 | 0.09183 | 10.46 | 41.68 |
| 10 | Dense Core Granule (GO:0031045) | 0.01874 | 0.09183 | 58.71 | 233.50 |

**Supplementary Table 3: GO Molecular Function analysis**

| **Index** | **Name** | **P-value** | **Adjusted p-value** | **Odds Ratio** | **Combined score** |
| --- | --- | --- | --- | --- | --- |
| 1 | Insulin Receptor Binding (GO:0005158) | 9.562e-7 | 0.00006789 | 207.95 | 2882.21 |
| 2 | Transmembrane Receptor Protein Tyrosine Kinase Adaptor Activity (GO:0005068) | 0.00008133 | 0.001835 | 191.08 | 1799.41 |
| 3 | Insulin-Like Growth Factor Receptor Binding (GO:0005159) | 0.00008133 | 0.001835 | 191.08 | 1799.41 |
| 4 | Hormone Activity (GO:0005179) | 0.0001034 | 0.001835 | 39.01 | 357.98 |
| 5 | Phosphatidylinositol Kinase Activity (GO:0052742) | 0.0002858 | 0.003680 | 95.49 | 779.20 |
| 6 | Neuropeptide Hormone Activity (GO:0005184) | 0.0003361 | 0.003680 | 87.52 | 700.01 |
| 7 | Neuropeptide Activity (GO:0160041) | 0.0003628 | 0.003680 | 84.02 | 665.56 |
| 8 | Protein Serine/Threonine Kinase Activity (GO:0004674) | 0.0004798 | 0.004258 | 13.01 | 99.40 |
| 9 | Phosphotyrosine Residue Binding (GO:0001784) | 0.0007215 | 0.005692 | 58.31 | 421.84 |
| 10 | Signaling Receptor Complex Adaptor Activity (GO:0030159) | 0.0009673 | 0.006530 | 49.97 | 346.83 |

**Supplementary Table 4: Protein-Protein Interaction analysis**

| **Index** | **Name** | **P-value** | **Adjusted p-value** | **Odds Ratio** | **Combined score** |
| --- | --- | --- | --- | --- | --- |
| 1 | IRS1 | 2.248e-12 | 3.461e-10 | 81.90 | 2196.65 |
| 2 | INSR | 9.963e-10 | 5.926e-8 | 50.73 | 1051.44 |
| 3 | AKT1 | 1.515e-9 | 5.926e-8 | 34.82 | 707.04 |
| 4 | IGF1R | 1.539e-9 | 5.926e-8 | 47.53 | 964.40 |
| 5 | PRKCZ | 1.007e-8 | 3.102e-7 | 52.88 | 973.66 |
| 6 | PRKCD | 1.871e-8 | 4.408e-7 | 47.45 | 844.41 |
| 7 | ERBB2 | 2.004e-8 | 4.408e-7 | 46.89 | 831.11 |
| 8 | CTNNB1 | 3.440e-8 | 6.148e-7 | 29.77 | 511.63 |
| 9 | PRKCE | 3.593e-8 | 6.148e-7 | 42.34 | 725.71 |
| 10 | ESR1 | 8.033e-8 | 0.000001237 | 20.47 | 334.48 |
